# Outcomes of the 2011 Botanical Nomenclature Section at the XVIII International Botanical Congress

**DOI:** 10.3897/phytokeys.5.1850

**Published:** 2011-07-27

**Authors:** James S. Miller, Vicki A. Funk, Warren L. Wagner, Fred Barrie, Peter C. Hoch, Patrick Herendeen

**Affiliations:** 1 The New York Botanical Garden, Bronx, NY, USA; 2 Smithsonian Institution, Washington DC, USA; 3 Missouri Botanical Garden, St. Louis, MO, USA; 4 Chicago Botanic Garden, Glencoe, IL, USA

**Keywords:** Algae, electronic publication, fungi, names, nomenclature, paleobotany, plants

## Abstract

The Nomenclature Section held just before the 18th International Botanical Congress in Melbourne, Australia in July 2011 saw sweeping changes to the way scientists name new plants, algae, and fungi. The changes begin on the cover: the title was broadened to make explicit that the Code applies not only to plants, but also to algae and fungi. The new title will now be the *International Code of Nomenclature of algae, fungi, and plants*. For the first time in history the *Code* will allow for the electronic publication of names of new taxa. In an effort to make the publication of new names more accurate and efficient, the requirement for a Latin validating diagnosis or description was changed to allow either English or Latin for these essential components of the publication of a new name. Both of these latter changes will take effect on 1 January 2012. The nomenclatural rules for fungi will see several important changes, the most important of which is probably the adoption of the principle of “one fungus, one name.” Paleobotanists will also see changes with the elimination of the concept of “morphotaxa” from the Code.

## Introduction

The Nomenclature Section (the Section), held in conjunction with the XVIII International Botanical Congress (IBC), met at the University of Melbourne from 18-22 July 2011. Some 200 delegates, most of them members of the International Association for Plant Taxonomy (IAPT), attended the Section, which meets once every six years on the occasion of the IBC. The Section is typically devoted to amending the *International Code for Botanical Nomenclature*, the detailed rules by which botanists name plants, fungi, and algae, but the meeting this July produced several momentous changes that will have major impacts on the way scientists communicate and organize information about these organisms.

The work of the Section is a broadly participatory international collaboration, intended to provide clear, fair rules that provide stability to the fundamental process of naming organisms and reflect changes in technology and in the science underpinning this process. Very briefly, proposals to amend the *Code* are submitted by any interested individual and are published in *Taxon* (the journal of IAPT) during the six years between Congresses. Prior to each IBC/Nomenclature Section, all members of IAPT and authors of proposals may vote on proposals to modify the Code. The proposed amendments are then discussed, debated, and voted on at the Nomenclature Section. All approved amendments are examined for conflicts by the Editorial Committee, which then finalizes the text for the new Code. Everyone who works on this process does so as a volunteer and the community can be proud that we have such an open process for making decisions on nomenclature.

## Results of the Nomenclature Section

One of the first changes involved altering the title of the *Code* to more accurately reflect its purview: following the meeting in Melbourne, it will be called the *International Code of Nomenclature of algae, fungi, and plants* (ICN). This change reflects efforts to ensure that the communities of biologists that study algae and fungi, which traditionally have been treated as plants, understand that this Code applies to their organisms. In addition, this explicit reference to algae and fungi on the cover signals the desire of the Section to continue to work with phycologists and mycologists to address their unique nomenclatural challenges within one code of nomenclature. This change lays the foundation for the acceptance of numerous rules that address the specific needs of various communities that study organisms that are quite different both in their biology and in the specific challenges they face in consistently applying names to their organisms.

After having rejected several similar proposals in several previous meetings, the Section approved a proposal to allow the names of new taxa to be considered effectively and validly published in specified types of electronic journals and books. Throughout the history of botany, effective publication of names has been accomplished only by hard-copy print materials. The new article in the Code, effective 1 January 2012, allows names to be accepted when they appear either in electronically published journals and books (e.g. Penev 2010) or in conventional printed material. As many universities and research institutions in the developing world cannot afford to subscribe to large numbers of journals, it is hoped that this will improve access for a greater number of the world’s taxonomists.

In another sweeping change, the long-standing historical requirement that all newly published names for plants, fungi, and algae be accompanied by a Latin description or diagnosis was significantly altered by the Section. Beginning 1 January 2012 names of new plants, algae, and fungi may now be published with a validating diagnosis or description that is written in either Latin or English. In an age where almost certainly 20% of the world’s plant species, and undoubtedly much greater percentages of fungi and algae, remain to be discovered, described, and named, this step will hopefully help taxonomists in their race to document biological diversity before it is lost to the deforestation and habitat degradation that threatens their extinction.

As molecular data have demonstrated that some large genera are polyphyletic, one of the most debated issues at the 2011 Nomenclature Section was related to the application of the generic name *Acacia*. The Section decided not to adopt any extraordinary exception into the Code and therefore approved the decision of the Vienna Congress to conserve an Australian species as the type of the genus.

Several changes in the Code have important consequences for the way names are applied for fungi. Historically, different names were applied to the sexual and vegetative forms of some fungi, but from now on, only a single name applies to each fungal species: a principle that has been articulated as “one fungus, one name.” In addition, starting 1 January 2012, names of new fungi will require the citation of a unique identifier issued by a recognized repository that will register the name.

Finally, the nomenclature of fossil plants (and fossil algae and fungi) will also see a significant change. Because organisms tend to fall apart after death and these dissociated fossilized parts are discovered and described independently, the naming of fossils can be complicated. Previous Codes have provided for “form-genera,” “organ-genera,” and most recently “morphotaxa” to accommodate different degrees of precision in understanding the taxonomic relationships of these fossils. The new Code clarifies for taxonomists that plant fossils are named (vs. fossil plants; Cleal and Thomas 2010) and it eliminates the concept of morphotaxa. In essence paleobotany has adopted the principle of “one fossil, one name,” analogous to the changes in mycological nomenclature. Efforts to assemble complete plants out of the separately named parts are important, but these whole plant reconstructions are hypotheses and are not governed by the principle of priority.
